# Indomethacin-Induced Gastric Ulcer in Rats: Gastroprotectivity of *Muscari neglectum* in Water

**DOI:** 10.3390/ph18010007

**Published:** 2024-12-24

**Authors:** Menekse Soydan, Gulnur Arabaci, Necati Utlu, Mesut Bünyami Halici, Esra Aktas Senocak, Metin Kiliçlioglu

**Affiliations:** 1 Institute of Natural Sciences, Sakarya University, Sakarya 54187, Türkiye; meneksesoydan54@gmail.com; 2 Department of Chemistry, Faculty of Science, Sakarya University, Sakarya 54187, Türkiye; 3 Department of Biochemistry, Faculty of Veterinary Medicine, Ataturk University, Erzurum 25240, Türkiye; nutlu@atauni.edu.tr (N.U.); mhalici@atauni.edu.tr (M.B.H.); 4 Horasan Vocational College, Ataturk University, Erzurum 25800, Türkiye; esenocak@atauni.edu.tr; 5 Department of Pathology, Faculty of Veterinary Medicine, Ataturk University, Erzurum 25240, Türkiye; metink@atauni.edu.tr

**Keywords:** *Muscari neglectum*, gastric ulcer, antioxidative, gastroprotective

## Abstract

**Background and Objectives:** The plant *Muscari Mill*. is employed in both raw and cooked forms for the treatment of gastric diseases, as an expectorant, and for the treatment of warts and the enhancement of urine. A review of the scientific literature revealed no studies investigating the effect of *Muscari neglectum* (MN) water extract on gastric diseases. The objective of this study was to determine the effect of a water extract of the MN plant on indomethacin-induced gastric ulcer in rats, using a series of biochemical (SOD, CAT, GSH and MDA levels) and histopathological parameters. **Methods:** 60 male Sprague Dawley rats were utilized for the purposes of evaluating the acute toxicity and gastric ulcer models, with a total of 36 rats employed for these experiments (n = 6). The rats were divided into six groups: intact; indomethacin; famotidine; indomethacin and MN (100, 200, 400 mg/kg). **Results:** The Gastric tissue examinations at biochemical, macroscopic and pathological levels showed that MN extracts effectively prevented indo-methacin-induced gastric mucosal damage. The 400 mg/kg dose exhibited the most effective antiulcer effect, with a 69% protective efficacy. This dose caused an increase in the SOD, CAT and GSH levels and a decrease in the MDA levels compared to the IND group. Furthermore, an LC-MS/MS analysis was conducted on the water extract of MN, resulting in the identification of 14 phenolic compounds. **Conclusions:** Biochemical analyses and histopathological examinations demonstrated that the water extract of MN exhibited a beneficial protective effect against gastric ulceration due to its high antioxidant content.

## 1. Introduction

Gastric ulcer is one of the most prevalent diseases worldwide, affecting approximately 10% of the global population. This disease is caused by a number of factors, including stress, an unhealthy diet, alcohol and cigarette addiction, infection with the bacterium *Helicobacter pylori*, and the overuse of nonsteroidal anti-inflammatory drugs (NSAIDs) such as indomethacin (IND). The disease occurs as a result of deterioration of the mucosal layer, which affects the superficial or deeper muscularis mucosa of the stomach, and reduces people’s quality of life and productivity [1,2].

The use of traditional plants in public and medicine is a practice that has been observed for many years. Medicinal aromatic plants (MAPs) are employed therapeutically for a range of diseases, yet their efficacy has not yet been sufficiently investigated to provide scientific evidence of their claimed biological activity. In order to ascertain the potential of these plants, it is necessary to conduct chemical analyses of their contents and constituents, as well as determine their biological, pharmacological and toxic effects [3,4].

Recent studies have shown that various herbs used in alternative medicine provide a general protective effect [5,6]. *M. neglectum* (MN) is one of this group of plants and it has been reported that its flowers, leaves, tubers and flower buds are consumed raw and cooked and can be used as gastritis and wart treatment, and a urine enhancer and expectorant [7]. The species *M. neglectum*, commonly known as grape hyacinth, has been the subject of numerous studies. These have revealed that it contains a range of chemical compounds, including homo-isoflavonoids [8], saponins, flavonoids, cardiotonic alkaloids, glycosides, terpenoids and steroids [9]. Additionally, it contains steroid compounds [10], succinic acid, uracil [11], aconitic acid, caffeic acid, quinic acid, fumaric acid, gentisic acid, apigenin and kaempferol [12,13], and is rich in antioxidant contents.

The objective of this study was to investigate the effects of water extracts of *M. neglectum*, a plant native to the Sakarya region with a high content of antioxidant components, on the protection of gastric tissue in rats. This was achieved by measuring the activity of basic biochemical parameters such as superoxide dismutase (SOD), catalase (CAT), glutathione (GSH) and malondialdehyde (MDA), as well as histopathological observations.

## 2. Results

### 2.1. Result of Acute Toxicity Study of the Water Extract of M. neglectum in Rats

*M. neglectum* (MN) given to test rats was found to be quite safe in an observational acute toxicity investigation; no toxic effects or fatalities were noted for 14 days. This demonstrated that the MN extract’s LD50 was higher than 2000 mg/kg.

### 2.2. Macroscopic Findings of Gastric Tissues

This study examined the anti-ulcer effects of famotidine (40 mg/kg, as a reference medicine) and various doses of *M. neglectum* plant extracts (100, 200, and 400 mg/kg) on experimental ulcers produced by indomethacin (IND).

**Figure 1:**
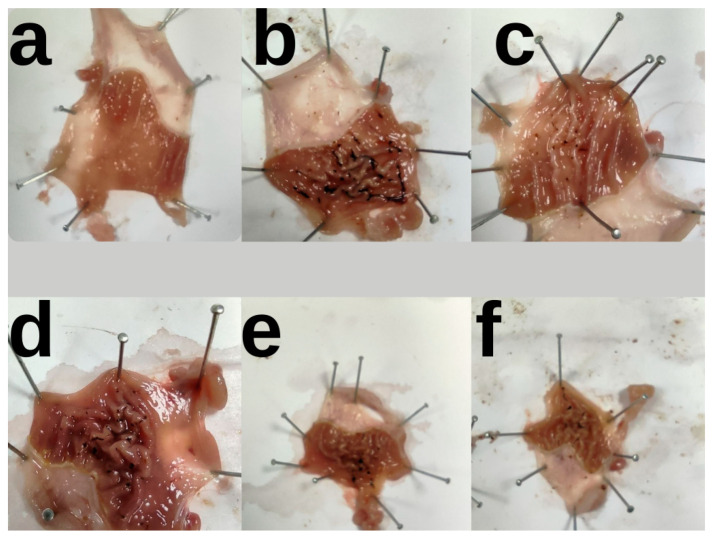
Results of gastric macroscopic pictures. (**a**), intact group; (**b**), indomethacin group; (**c**), reference group (40 mg/kg of famotidine); (**d**–**f**), treatment groups (100, 200 and 400 mg/kg b.w. of *M. neglectum*, respectively).

All doses of MN extracts were observed to prevent stomach mucosal damage caused by indomethacin (*p* < 0.001), with the 400 mg/kg dose exhibiting the highest antiulcer efficacy with 69% (Table 1). Famotidine, which was utilized as a reference, had a similar impact (Figure 1). In the tables and figures, all values are displayed as mean ± standard deviation.

### 2.3. Histopathologic Findings of Gastric Tissues

**Figure 2:**
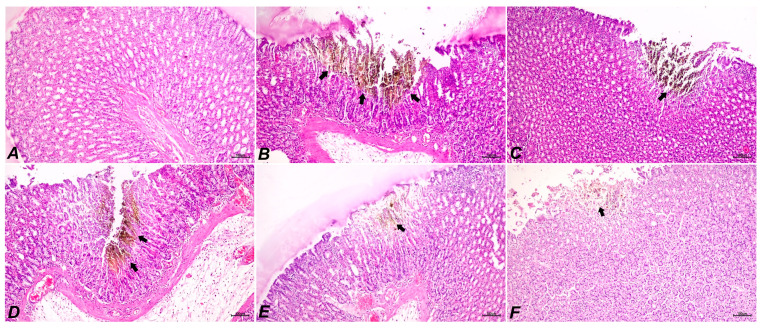
Results of gastric morphology and H&E staining. Results of gastric H&E staining section 100 µm. (**A**), intact group; (**B**), indomethacin group; (**C**), reference group (40 mg/kg of famotidine); (**D**–**F**), treatment groups (100, 200 and 400 mg/kg b.w. of *M. neglectum*, respectively). Necrotic area (arrows). Scale bar: 100 µm.

Intact group: a normal histologic structure was found in the gastric tissues after histopathologic analysis (Figure 2A).

Indomethacin group: Histopathologic analysis of gastric tissues showed that the tunica mucosa’s lamina epithelium was severely eroded and ulcerated, and that this erosion spread to the lamina propria. The interstitial spaces showed severe epithelial degradation and necrosis, cell infiltration, and hemorrhage foci. There was considerable hyperemia in the arteries and severe edema in the submucosa (Figure 2B).

Reference group treated with 40 mg/kg famotidine: Histopathological examination of gastric tissues revealed very mild erosion of the lamina epithelium, along with degeneration and necrosis of the epithelium. Additionally, moderate hyperemia of the vessels in the lamina propria was observed (Figure 2C).

IND+100MN group: In the histopathological examination of gastric tissues, moderate erosions were noted in the lamina epithelium extending to the lamina propria. There was also moderate degeneration and necrosis of the epithelium, along with severe edema observed in the submucosa (Figure 2D).

IND+200MN group: Histopathological examination of gastric tissues revealed mild erosion of the lamina epithelium, degeneration and necrosis of the epithelium, and moderate hyperemia in the vascular structures. Additionally, mild edema was observed in the submucosa (Figure 2E). A statistically significant difference (*p* < 0.05) was detected when compared to the indomethacin group.

IND+400MN group: Histopathological examination of gastric tissues revealed very mild erosion of the lamina epithelium, degeneration and necrosis of the epithelium, and mild hyperemia of the vessels (Figure 2F). A statistically significant difference (*p* < 0.05) was found when compared to the indomethacin group. The histopathological findings are summarized in Table 2.

### 2.4. Biochemical Parameters

The formation of indomethacin-induced ulcers is significantly influenced by free oxygen radicals. Consequently, this study aimed to ascertain the impact of indomethacin on the antioxidant defense mechanism of MN. This was achieved through the analysis of GSH, MDA, SOD and CAT enzyme activities (Table 3). The results of the analysis of SOD, CAT enzyme activities, and GSH and MDA levels in rat gastric tissues are presented in Table 3.

While there was no significant difference in SOD and CAT activities between the intact group and the IND+100MN and IND+200MN groups (*p* > 0.05), a significant difference was observed when compared with the IND, IND+FAM, and IND+400MN groups (*p* < 0.0001).

No significant difference was observed in the SOD activities of the IND group when compared with the IND+100MN and IND+200MN groups (*p* > 0.05). However, a significant difference was noted when the IND group was compared with the IND+FAM and IND+400MN groups (*p* < 0.0001). A significant difference was observed when the CAT activities of the IND group were compared with those of the other groups (*p* < 0.0001). This indicated that the most effective MN dose was 400 mg/kg (*p* < 0.0001).

No notable discrepancy was identified in the GSH concentration of the intact group when compared with IND+FAM, IND+200MN, and IND+400MN (*p* > 0.05). However, a statistically significant divergence was observed in the IND and IND+100MN groups (*p* < 0.0001).

In comparison to the IND group, the IND+FAM, IND+200MN, and IND+400MN groups exhibited elevated GSH levels. The administration of 200 and 400 mg/kg doses of MN, whose effect was tested, resulted in a significant increase in the GSH levels (*p* < 0.0001). However, no significant difference was observed between the MN-treated and the reference groups (*p* > 0.05).

While no significant difference was observed between the MDA levels of the intact group and those of the IND+FAM and IND+200MN groups (*p* > 0.05), this difference was found to increase the MDA levels in the IND and IND+100MN groups and to decrease these in the IND+400MN group (*p* < 0.0001). The 100 mg/kg dose of MN, which was subjected to testing, demonstrated no impact on MDA levels. In contrast, the 200 and 400 mg/kg doses exhibited a comparable effect with the reference group, resulting in a reduction in MDA levels (*p* < 0.0001).

Significant differences were observed in SOD and CAT activities when the IND group, IND+FAM, and IND+400MN groups were compared (*p* < 0.0001).

### 2.5. LC-MS/MS Analysis

The LC-MS/MS analysis of the phenolic compounds present in the water extracts of the above-ground parts of *M. neglectum*, as used in the experiment, is presented in Table 4. The analysis revealed the presence of fumaric acid, protocatechuic acid, phloridzindyhrate and myricetin compounds in significant quantities, while acetohydroxamic acid, syringic acid, resveratrol, kaempferol, gallic acid, 4-hydroxybenzoic acid, caffeic acid, salicylic acid, quercetin and luteolin compounds were also identified in lesser amounts. It was observed that some of the components identified in the studies of MN using different solvents were consistent with those identified in our study, while others were distinct. This discrepancy is postulated to be attributable to the utilization of disparate plant parts and solvents. Phenolic compounds have been demonstrated to possess anti-cancer, anti-inflammatory, anti-ulcer and antioxidant properties with respect to human health [13].

## 3. Discussion

Medicinal aromatic plants (MAPs) have been shown to have antioxidant properties due to the presence of phenolic compounds in their composition [13,14,15,16,17,18,19,20,21,22,23,24]. In this study, the water extract of the aerial parts of MN collected in Sakarya was analyzed by LC-MS/MS for antioxidant molecules and the presence of significant amounts of fumaric acid, protocatechuic acid, fluoridzindihrate and myricetin compounds was revealed. The extract also contained trace amounts of acetohydroxamic acid, syringic acid, resveratrol, kaempferol, gallic acid, 4-hydroxybenzoic acid, caffeic acid, salicylic acid, quercetin and luteolin. Eroğlu and Dogan [12] identified quinic acid, fumaric acid, gentisic acid, caffeic acid, kaempferol and apigenin in the LC-MS/MS analysis of MN ethanolic extract of the aerial parts collected from the Van region. Furthermore, Mahomoodally et al. [13] identified quinic acid and fumaric acid as the dominant compounds in the LC-MS/MS analysis of MN methanol flower extracts collected from the Konya region. A comparison of the present study with the findings of Eroglu and Dogan [11] and Mahomoodally et al. [13] revealed the presence of distinct phenolic compounds in the MN plant. The presence of different phenolic compounds in the same plant suggests that this may be attributed to the solvent or soil structure of the region.

Prior research has demonstrated that protocatechuic acid (PCA) exerts a protective effect against gastrointestinal oxidative damage, neurotoxicity, nephrotoxicity and hepatotoxicity. Consequently, PCA is regarded as an effective antioxidant and anti-radical protector [4,15].

Park et al. [15] investigated the protective effect of myricetin, a flavonoid, against gastric damage and observed that it increased total glutathione (GSSG/GSH) and prostaglandin E levels, and SOD and cyclooxygenase-1 (COX-1) activity, and decreased MDA levels in gastric tissues.

The therapeutic properties of fumaric acid (FA) against Cd-induced toxicity in rats were determined by a significant decrease in lipid peroxidation and an increase in the GSH levels, GPx, SOD and CAT activities. It was proposed that this effect can protect cellular membrane integrity against free radicals and lipid peroxidation products due to the antioxidant properties of FA [17].

In their study, Patel et al. [18] demonstrated that locally applied dimethyl fumarate is an effective treatment for gastric ulcers caused by ethanol.

Shakya et al. [19] demonstrated that monomethyl fumarate, a constituent of methanolic extracts of *Fumaria indica*, is an efficacious gastroprotective agent against gastric ulcers and other pathologies precipitated by chronic and severe stress.

It has been demonstrated that caffeic acid has the capacity to enhance gastric mucosal integrity [20], while kaempferol and apigenin have been shown to exert anti-ulcerogenic effects [21,22]. These observations have been attributed to the antioxidant properties of these compounds.

Earlier studies have indicated that resveratrol can impede the proliferation of *Helicobacter pylori*, a bacterium that is a primary etiological agent of gastric ulcers, gastritis, and gastric cancer. Additionally, resveratrol has been demonstrated to inhibit the growth of breast cancer cells [23].

The aforementioned studies have predominantly indicated that phenolic compounds possess protective properties against gastric damage. Similarly, our study demonstrated that the water extract of the above-ground parts of the MN plant, which contains these phenolic components, enhanced the level of antioxidant enzymes through a synergistic effect, reduced the oxidant factor, and exhibited a protective effect against gastric damage.

It has been found that in vivo studies on the MN plant in the literature are limited. In this study, it was determined for the first time in the literature that the water extract of MN plant showed a gastroprotective effect. The biochemical, macroscopic and histopathological examination of the gastric tissue in the model system in this study demonstrated that all doses of water extracts of the MN plant effectively prevented indomethacin-induced gastric mucosal damage. The 400 mg/kg dose exhibited the most pronounced antiulcer effect, with a protective efficacy of 69%. Biochemical evaluation of oxidative stress enzymes revealed an increase in the SOD, CAT and GSH levels and a decrease in the MDA levels in the indomethacin-induced gastric ulcer group.

In a study by Eroğlu and Dogan [12], the protective effects of an ethanolic extract of the aerial parts and bulb of MN at a dose of 400 mg/kg were investigated on carbon tetrachloride (CCl_4_)-induced gastric damage in rats. The findings indicated that MN caused a statistically significant decrease in the malondialdehyde (MDA) levels and an increase in the antioxidant parameters. In the present study, the effects of three different doses of MN water extract (100, 200, 400 mg/kg) were investigated in order to ascertain their efficacy in protecting against gastric damage caused by indomethacin. The most effective dose was determined to be 400 mg/kg, which demonstrated effects that were comparable to those observed with famotidine (reference). In comparison to the 35-day study conducted by Eroğlu and colleagues, our study demonstrated that the water extract of MN aerial parts exhibited a protective effect against indomethacin-induced gastric damage. This was evidenced by a reduction in the MDA levels and an increase in the antioxidant parameters within six hours. Our findings align with those of Eroğlu and Doğan [12], yet our study additionally revealed that the water extract demonstrated a protective impact against indomethacin-induced gastric ulcers in rats within a remarkably brief timeframe of six hours. Moreover, these observations were corroborated by pathological assessments.

## 4. Materials and Methods

### 4.1. Plant and Animal Material

*M. neglectum* (MN) was harvested from Sakarya province (40°44′41.2″ N 30°19′47.0″ E) in April 2022. The identification of the plant was conducted by the Department of Biology at Sakarya University’s Faculty of Science, and the plant material (M. Sağiroğlu 7068) was catalogued in the Sakarya University Herbarium (SAKU). The MN was desiccated in a shaded environment at room temperature and kept in the dried environment until the commencement of this study.

Rats were used as the animal materials with the authorization of Atatürk University Animal Experiments Local Ethics Committee (No. 2022/213). A total of 60 male Sprague Dawley rats, with a weight range of 250–300 g, were supplied by the Atatürk University Medical Experimental Application and Research Center Laboratories. The rats were housed in a controlled environment with a 12 h light/12 h dark cycle, at a temperature of 25 ± 1 °C, and a humidity level of 55–60%. The rats were fed ad libitum with standard rat food and water.

### 4.2. Phytochemical Screening and Antioxidant Activity Measurement

#### Plant Extraction and LC-MS/MS Analysis

The aerial parts of *M. neglectum* were dried, ground and extracted with distilled water at 60 °C for 30 min. The extract was then lyophilized (Bıobase-BK-FD10P) and the percentage yield value was determined. The phenolic content of the aqueous extract was determined by liquid chromatography with tandem mass spectrometry (LC-MS/MS) (Shimadzu-8030) [25].

### 4.3. Acute Toxicity Test of Crude Plant Extract

In this section, a total of 24 rats were used as animal materials and four separate study groups (intact group, 500 mg/kg, 1000 mg/kg and 2000 mg/kg *M. neglectum* extracts groups) consisting of six rats each were established. Prior to the administration of *M. neglectum*, the rats were fasted for a period of 24 h. Thereafter, the toxicity and mortality-related behaviors of the rats were observed by periodic monitoring at 24 h intervals for a period of 14 days [26].

### 4.4. Experimental Protocol and Indomethacin-Induced Ulcer Model

Six rats were randomly assigned to each of the following six groups: intact, reference (Famotidine; Famodin^®^ tablets, 20 mg/tablet; Sandoz Drug, Istanbul, Turkey), control (Indomethacin; Endol^®^ capsules, 25 mg/capsule; Deva Drug, Istanbul, Turkey), and treatment (100, 200, and 400 mg/kg *M. neglectum*). The rats in the reference group received 40 mg/kg of famotidine orally, while the rats in the treatment groups received dosages of 100, 200, and 400 mg/kg of *M. neglectum* after a 24 h fast to prevent ulcer formation. All rats, with the exception of the intact group, received a gastric gavage of 25 mg/kg of indomethacin five minutes after the application [27]. The rats were beheaded under moderate sevoflurane anesthesia following the six-hour treatment. Every animal’s stomach was opened along the big quarter, cleaned with saline, and then studied under a microscope. The stomach tissues of every rat in the experimental groups were then preserved in 10% formalin solution for histopathological analysis and in a deep freezer at −20 °C for biochemical analysis.

### 4.5. Biochemical Analysis of Gastric Tissues

After the gastric tissues were lysed, diluted 1/9 with phosphate buffer (pH:7.4), and homogenized (using a TissueLyser LT (Qiagen, Hilden, Germany) at 50 Hz for 1 min), the homogenates were centrifuged at 5000× *g* for 5 min at +4 °C, and the supernatants were separated. Using commercial kits (BT Lab., Shanghai, China), the ELISA method (sandwich enzyme-linked immunoassay) was used to measure the levels of SOD, CAT enzyme activities, GSH, and MDA in the supernatants.

#### 4.5.1. Macroscopic Examination of Gastric Tissues

The test rats’ stomach tissues were opened, pictures were obtained, and the Stereo Investigator program was used to compute the ulcer regions and anti-ulcer effect (%) [28].

#### 4.5.2. Histopathologic Examination of Gastric Tissues

Tissue samples collected at the conclusion of the examination were embedded in paraffin blocks following standard tissue follow-up techniques and preserved in a 10% formaldehyde solution for 48 h. Each block was divided into slices that were 4 m thick. The tissues that were ready for histopathologic analysis were then stained with hematoxylin-eosin (HE) and observed under a light microscope (Olympus BX 51, Olympus Life Science, Tokyo, Japan). Based on histopathologic characteristics, the sections were assessed as mild (+), moderate (++), severe (++++), and absent (−).

### 4.6. Statistical Analysis

All collected data were analyzed using the SPSS 13.0 package, and data were assessed by considering *p* < 0.05 to be significant. In biochemical measurements, the “One-way Analysis of Variance (ANOVA)” test was used to explain statistical variations and significance levels. Multiple comparisons were examined using Tukey’s post hoc test. In histopathologic exams, the Duncan test was employed to compare groups. Group interaction was assessed using the non-parametric Kruskal–Wallis test, while group differences were assessed using the Mann–Whitney U test.

## 5. Conclusions

In consideration of the findings, it was determined that the lethal dose of water extract of *M. neglectum* in rats exceeded 2000 mg/kg. Biochemical and histopathological observations indicated that a dose of 100 mg/kg of *M. neglectum* did not provide significant protection against gastric ulcers. However, doses of 200 and 400 mg/kg were observed to exert a protective effect, as evidenced by a reduction in the malondialdehyde (MDA) levels and an increase in the superoxide dismutase (SOD), catalase (CAT) and glutathione (GSH) levels. It can be concluded that this observed effect is at least partly attributable to the phenolic components present in MN. In light of these findings, further toxicological and clinical studies will be conducted in the future with a view to elucidating the safety profile of MN more clearly and facilitating its use as an alternative treatment.

## Figures and Tables

**Table 1:** Ulcer area and anti-ulcer effects in test rats.

Groups	Dose (mg/kg)	n	Ulcer Area (mm^2^)	Anti-Ulcer Effect (%)
INTACT		6	0.0 ± 0.0 ^d^	-
IND	25	6	23.16 ± 3.76 ^a^	-
IND+FAM	40	6	7.0 ± 0.07 c	67%
IND+100MN	100	6	14.84 ± 6.5 ^b,c^	36%
IND+200MN	200	6	10.52 ± 3.83 ^b,c^	54%
IND+400MN	400	6	7.08 ± 1.97 ^c^	69%

Results are shown as the mean (mean ± SD) of measurements across rat groups. It was accepted that there was no statistically significant difference between the values shown with the same letter or letters in the same column compared to the IND control group (*p* ˃ 0.05). It was accepted that there was a significant difference between the values shown with different letters (*p* ˂ 0.001). Tukey’s test was applied for multiple comparisons. Data are represented as means ± SD (*n* = 6). IND: Indomethacin; IND+FAM: Indomethacin + Famotidine, IND+100MN: Indomethacin + 100 mg/kg *M. neglectum*; IND+200MN: Indomethacin + 200 mg/kg *M. neglectum*; IND+400MN: Indomethacin + 400 mg/kg *M. neglectum.*

**Table 2:** The scoring of histopathologic findings in gastric tissues.

	Erosion Ulceration of the Lamina Epithelium	Epithelium Degeneration and Necrosis	Hyperemia of the Veins	Edema in Submucosal
INTACT	−	−	−	−
IND	++++	++++	++++	++++
IND+FAM	+	++	++	−
IND+100MN	+++	+++	++++	++++
IND+200MN	++	++	+++	+
IND+400MN	+	+	++	−

IND: Indomethacin; IND+FAM: Indomethacin + Famotidine, IND+100MN: Indomethacin + 100 mg/kg *M. neglectum*; IND+200MN: Indomethacin + 200 mg/kg *M. neglectum*; IND+400MN: Indomethacin + 400 mg/kg *M. neglectum.*

**Table 3:** SOD, CAT enzyme activities and GSH and MDA levels analyzed in rats’ gastric tissues.

Groups	Dose (mg/g)	n	SOD (ng/mg Wet Tissue)	CAT (ng/mg Wet Tissue)	GSH (mg/g Wet Tissue)	MDA (nmol/mg Wet Tissue)
INTACT		6	1.26 ± 0.09 ^b^	16.83 ± 0.14 ^c^	113.16 ± 0.01 ^b^	0.59 ± 0.11 ^b^
IND	25	6	0.58 ± 0.2 ^a^	6.61 ± 0.01 ^a^	49.81 ± 0.01 ^a^	1.13 ± 0.11 ^a^
IND+FAM	40	6	2.15 ± 0.1 ^c^	33.99 ± 0.1 ^e^	137.5 ± 0.1 ^b^	0.55 ± 0.08 ^b,c^
IND+100MN	100	6	1.05 ± 0.15 ^a,b^	14.71 ± 0.4 ^b,c^	71.46 ± 0.14 ^a^	1.05 ± 0.17 ^a^
IND+200MN	200	6	1.13 ± 0.05 ^a,b^	18.21 ± 0.14 ^c^	124.52 ± 0.11 ^b^	0.56 ± 0.07 ^b,c^
IND+400MN	400	6	3.22 ± 0.20 ^d^	26.49 ± 0.08 ^d^	134.51 ± 0.13 ^b^	0.31 ± 0.09 ^c^

Results are shown as the mean (mean ± SD) of measurements across rat groups. It was accepted that there was no statistically significant difference between the values shown with the same letter or letters in the same column compared to the IND control group (*p* ˃ 0.05). It was accepted that there was a significant difference between the values shown with different letters (*p* ˂ 0.001). Tukey’s test was applied for multiple comparisons. Data are represented as means ± SD (n = 6). IND: Indomethacin, FAM: Famotidine, MN: *M. neglectum*, SOD: Superoxide dismutase, CAT: Catalase, GSH: Glutathione, MDA: Malonaldehyde.

**Table 4:** Quantitative analysis of phytochemicals in the water extract of *M. neglectum* aerial part by LC-MS/MS.

	Analytes	R.Time	*m*/*z*	Compound Content (mg/g of Dry Extract)
1	Acetohydroxamic Acid (+)	0.460	76.15 > 58.00	0.0066244
2	Catechinhyrate (+)	2.482	291.00 > 139.00	ND
3	Vanilic Acid (+)	2.853	168.95 > 65.00	ND
4	Syringic Acid (+)	2.973	199.00 > 140.00	0.00575919
5	Thymoquinone (+)	3.338	165.00 > 137.10	ND
6	Resveratrol (+)	3.632	229.00 > 135.00	0.00322667
7	Myricetin (+)	3.725	319.00 > 153.00	0.01061646
8	Kaempferol (+)	4.222	287.00 > 153.00	0.00270288
9	Fumaric Acid (−)	0.859	115.30 > 71.20	0.30281874
10	Gallic Acid (−)	1.483	169.10 > 124.90	0.00455979
11	Protocatechuik Acid (−)	2.060	152.80 > 108.00	0.16369329
12	4-Hydroxybenzoic Acid (−)	2.550	137.20 > 93.00	0.00475702
13	Caffeic Acid (−)	2.850	178.80 > 134.90	0.00280178
14	Salisilik Acid (−)	3.480	137.20 > 93.10	0.0015531
15	Phloridzindyhrate (−)	3.523	435.30 > 272.90	0.02561198
16	2-Hydoxycinamic Acid (−)	3.553	162.80 > 118.90	ND
17	Oleuropein (−)	3.587	539.20 > 275.10	ND
18	2-hyroxy1,4 naphthaquinone (−)	3.654	172.80 > 144.90	ND
19	Naringenin (−)	3.960	271.20 > 150.90	ND
20	Silymarin (−)	4.000	481.10 > 124.90	ND
21	Quercetin (−)	4.000	300.80 > 150.80	0.00627964
22	Luteolin (−)	4.122	285.20 > 133.00	0.00278937
23	Alizarin (−)	4.552	238.80 > 211.00	ND
24	Curmin (−)	4.605	367.00 > 216.90	ND

## Data Availability

The original contributions presented in the study are included in the article, further inquiries can be directed to the corresponding authors.

## Abbreviations

MN, *Muscari neglectum*; FAM, famotidine; IND, indomethacin; SOD, superoxide dismutase; GSH, reduced glutathione; MDA, malondialdehyde; COX-1, cyclooxygenase-1; CCl_4_, carbon tetrachloride; FA, fumaric acid; PCA, protocatechuic acid.
